# Sex-specific hormone changes during immunotherapy and its influence on survival in metastatic renal cell carcinoma

**DOI:** 10.1007/s00262-021-02882-y

**Published:** 2021-02-28

**Authors:** Gennadi Tulchiner, Renate Pichler, Hanno Ulmer, Nina Staudacher, Andrea Katharina Lindner, Andrea Brunner, Bettina Zelger, Fabian Steinkohl, Friedrich Aigner, Wolfgang Horninger, Martin Thurnher

**Affiliations:** 1 Department of Urology & Immunotherapy Unit, Medical University Innsbruck, Anichstrasse 35 and Innrain 66a, 6020 Innsbruck, Austria; 2 Department of Medical Statistics, Informatics and Health Economics, Medical University Innsbruck, Schoepfstraße 41, 6020 Innsbruck, Austria; 3 Department of Pathology, Medical University Innsbruck, Muellerstraße 44, 6020 Innsbruck, Austria; 4 Department of Radiology, Medical University Innsbruck, Anichstrasse 35, 6020 Innsbruck, Austria

**Keywords:** Renal cell carcinoma, Checkpoint inhibition, Sex hormones, Estrogen, Luteinizing hormone/follicle-stimulating hormone, Immunotherapy

## Abstract

Renal cell carcinoma (RCC) is a highly vascularized and immunogenic tumor, being an ideal candidate for checkpoint blockade-based immunotherapy. Accordingly, checkpoint inhibitors have demonstrated clinical efficacy in patients with metastatic RCC (mRCC). Sex-specific differences in cancer immunotherapy may be explained by the interaction of sex hormone signaling, genetic and environmental factors, affecting the innate and adaptive immune response in men and women in different ways. The aim of this prospective study was to monitor for the first time changes in sex hormones including luteinizing hormone (LH), follicle-stimulating hormone (FSH), LH/FSH ratio and 17-ß-estradiol (E2) in 22 mRCC patients (12 male and 10 female) receiving nivolumab therapy. In contrast to female patients, male patients showed a significant increase in E2 (*p* = 0.006) and LH/FSH ratio (*p* = 0.013) from the beginning of nivolumab therapy to week 12 of follow-up. Moreover, survival analysis revealed a significant negative association between LH/FSH ratio and progression-free survival (PFS) (*p* = 0.022) as well as between therapy response (*p* = 0.009) in males compared to females at interim evaluation (week 6/8). Our findings may therefore be the first reference to sex hormone changes during immunotherapy.

**Supplementary Information:**

The online version contains supplementary material available at (10.1007/s00262-021-02882-y)

## Introduction

Renal cell carcinoma (RCC) is one of the most immune-responsive cancers in humans [1]. Immunotherapy with interleukin-2 (IL-2) and/or interferon alpha (IFN-α) was thought to increase and mobilize tumor-infiltrating lymphocytes (TILs), facilitating tumor regression. However, this form of cytokine-based immunotherapy showed only few durable complete remissions [2–5]. Modified tumor cells [6] or tumor antigen-loaded dendritic cells (DCs) [7] were used to activate antitumor T cell responses in vivo [1]. DC vaccination, which counts as a personalized treatment modality, was found to induce antigen-specific [7] as well as antigen-independent immune responses [8] and to maintain quality of life [9].

The introduction of immunotherapy in RCC revolutionized cancer therapy with the use of immune checkpoint inhibitors. Nivolumab is a fully human IgG4 antibody specific for PD-1, which blocks suppressive PD-1 signaling induced by its ligands and thereby restores antitumor T cell immunity [10]. According to the Checkmate 025 study [11], nivolumab was the first immune checkpoint inhibitor that was approved for second line treatment of patients with mRCC based on significantly better overall survival (OS). Furthermore, nivolumab demonstrated a higher objective response rate (ORR), fewer grade 3 or 4 treatment-related adverse events (AEs) and improved quality of life compared with the mTOR inhibitor everolimus [12]. Moreover, nivolumab demonstrated an OS improvement versus everolimus across various subgroups such as: International Metastatic RCC Database Consortium (IMDC) risk groups; age; one, two or more sites of metastases; number and duration of prior therapy; bone, liver and lung metastases and finally the type of prior therapy (sunitinib, pazopanib or IL-2 therapy) [13]. Further, novel combination therapies, such as nivolumab plus ipilimumab (CTLA-4-inhibitor), avelumab (PD-L1-inhibitor) plus axitinib, a tyrosine kinase inhibitor (TKI) and finally pembrolizumab (PD-1-inhibitor) plus axitinib, showed improvement in first-line mRCC therapy [14–17].

In addition to immunogenicity and vascularization, hormone-dependence is another feature of RCC [18]. Although the role of sex hormones in RCC development remains controversial, it is strongly implied by the higher incidence of RCC in males, who are affected twice as often [18]. Sex is a known factor that influences both innate and adaptive immune response [19] including sex-specific differences in immunotherapy response [20]. These findings are corroborated by increasingly reported sex disparities in response to immunotherapy in a variety of tumor types. However, contradictory results have also been reported. No significant correlation between sex and response to immunotherapy could be demonstrated in two recently published meta-analyses that included different cancer types and compared 9322 men with 4399 women [21] and 12,674 men with 7025 women, respectively [22]. On the contrary, other reports showed that female sex is an unfavorable predictive factor for response to immunotherapy [19, 23]. Moreover, a significant survival benefit was reported for men treated with anti CTLA-4 or anti-PD-1 therapies in a large meta-analysis of 20 randomized clinical trials including 11,351 patients with advanced or metastatic cancer [24], whereas the role of gender was examined in these studies and sex hormones have hardly been assessed. Sex hormones are considered responsible for the differences between male and female immune responses. For instance, estrogens are known to increase the production of immunoglobulins [25–27]. Androgens including dihydrotestosterone and testosterone have been reported to suppress immune activity [28]. Regulatory T cells (T_reg_ cells) increase with high estrogen and decrease with low estrogen levels [29]. Moreover, low estrogen levels promote T helper (Th) differentiation toward Th1, while high doses of estrogen favors the Th2 phenotype [30]. Increased expression of PD-1 has also been shown to be mediated by estrogen [29]. Although the regulatory role of sex hormones in the immune system is well established, they have not been assessed during immunotherapy of mRCC.

In the present study, we have therefore monitored hormones of pituitary–gonadal axis in 22 mRCC patients during anti-PD-1 immunotherapy with nivolumab, evaluating their relation to immunotherapy response and oncological prognosis.

## Patients and methods

### Study populations and data collection

We performed a retrospective analysis of a prospectively collected database of 22 patients with mRCC undergoing immunotherapy with the PD-1 inhibitor nivolumab at our department between May 2016 and January 2020. The study was approved by a local institutional review board (ethics committee study number: 1202/2018). In April 2016, nivolumab was approved for intravenous (IV) administration of a 3 mg/kg dose once every 2 weeks in 12-week treatment cycles. Modifications of the dosage regimen to a flat dose of 240 mg as single dose every two weeks and 480 mg every four weeks were approved in September 2016 and June 2018, respectively [31].

Data were collected from patients’ electronic medical records including demographic information, smoking behavior, sequence of systemic therapy, duration of treatment, best response to PD-1 inhibition, date of progression, date of death or last follow-up at our outpatient department and detailed RCC histology. Quantitative serum measurements of LH, FSH, LH/FSH ratio, E2, testosterone and prolactin were performed enzyme-linked immunosorbent assay (ELISA) at three different landmarks. The first measurement was at the beginning of nivolumab therapy (baseline/week 0). Due to a regimen change from 240 mg every two weeks to 480 mg every four weeks, the landmark analyses were performed at 6 and 8 weeks, respectively, (interim analysis/week 6/8). The final evaluation was carried out at week 12 (final analysis/week 12). Sex-specific reference values for men were 0.8–7.6 U/L for LH, 1.6–20.4 U/L for FSH, 11–43 ng/L for E2, 1.70–4.90 µg/L for testosterone and 2.5–17.0 µg/L for prolactin. According to medical history, all female patients were confirmed to be postmenopausal without ongoing hormone replacement therapy. Female reference values were 11.3–39.8 U/L for LH, 35.0–150.0 U/L for FSH, < 13–65 ng/L for E2, 0.00–0.40 µg/L for testosterone and 1.9–25.0 µg/L for prolactin.

Furthermore, we performed an exact calculation according to IMDC risk model for mRCC on the basis of the following values: hemoglobin < lower limit of laboratory reference range, corrected calcium > 10.0 mg/dL (2.4 mmol/L), platelets > upper limit of normal, absolute neutrophil count (ANC) > upper limit of normal, Karnofsky Performance Status (KPS) < 80%, and time from diagnosis to systemic treatment < 1 year before starting first-line therapy [32].

Response and progression were determined via computed tomography (CT) at week 12 using the Response Evaluation Criteria in Solid Tumor version 1.1 (RECIST 1.1) criteria [33] by two uro-radiologists (F.A. and F.S.). Stable disease (SD), partial remission (PR) or complete remission (CR) as primary response were necessary for treatment continuation. ORR was defined as the number of patients with complete response or partial response divided by the total number of patients. Best overall response was defined as the best response from therapy start to progression or subsequent therapy, whichever occurred first. PFS and OS were defined as time from therapy initiation to death from all causes for OS and to progression, treatment discontinuation or tumor-specific death for PFS, censored at last follow-up for those still alive or who have not progressed.

### Statistical analysis

Statistical analyses were carried out using SPSS version 24 (IBM Corp. Chicago, IL, USA). Graphs and Figures were made using SPSS version 24 (IBM Corp. Chicago, IL, USA) and GraphPad Prism 8.4.1. Descriptive statistics for continuous variables are reported as means with standard deviation if the data showed normal distribution as well as medians and interquartile ranges (IQR), if the data failed the test for normality. Kolmogorov–Smirnov and Shapiro–Wilk tests were used to test the data for normal distribution. Categorical variables are reported as frequencies and percentages. Continuous variables between patient groups were compared by the Student’s t-test in case of normal distributed data and the Mann–Whitney U test, in case of data that deviated from normal distribution. ANOVA and the paired t-test were used for repeated measurements to show changes in LH, FSH, LH/FSH ratio, E2, testosterone and prolactin between baseline and after 12 weeks of treatment, if the data passed the tests for normality. If data do not allow parametric testing Kruskal–Wallis and Friedman tests were applied. The associations between sex hormones and response to therapy were analyzed by an independent t-test, if normal distributed and the Mann–Whitney U test, if the normality could not be shown. All statistical tests were two-sided, and overall statistical significance was defined as *p* < 0.05*.* Bonferroni method was applied to correct for multiple comparisons. Regarding survival and response, which were separately assessed in females and males, a *p* < 0.025 was considered to indicate statistical significance. Regarding the repeated measurements with additional evaluation at two evaluation time points (interim/final evaluation) a *p* < 0.0125 was chosen.

To examine the sex hormone status and its associations with survival, patients were split into a high and low group using the gender-specific median as cutoff level. Correlations between hormonal changes and OS or PFS were analyzed by applying Kaplan–Meier survival analysis, the cutoff was dichotomized in low vs. high at the median of 0.69 for the male population and at the median of 0.36 for the female population.

For the LH/FSH ratio, receiver operating characteristic (ROC) curves were plotted, and the area under the curve (AUC) was calculated. The optimal cutoff value for the LH/FSH ratio at week 6/8 was determined as the value with the highest Youden index.

## Results

### Baseline characteristics

**Fig. 1:**
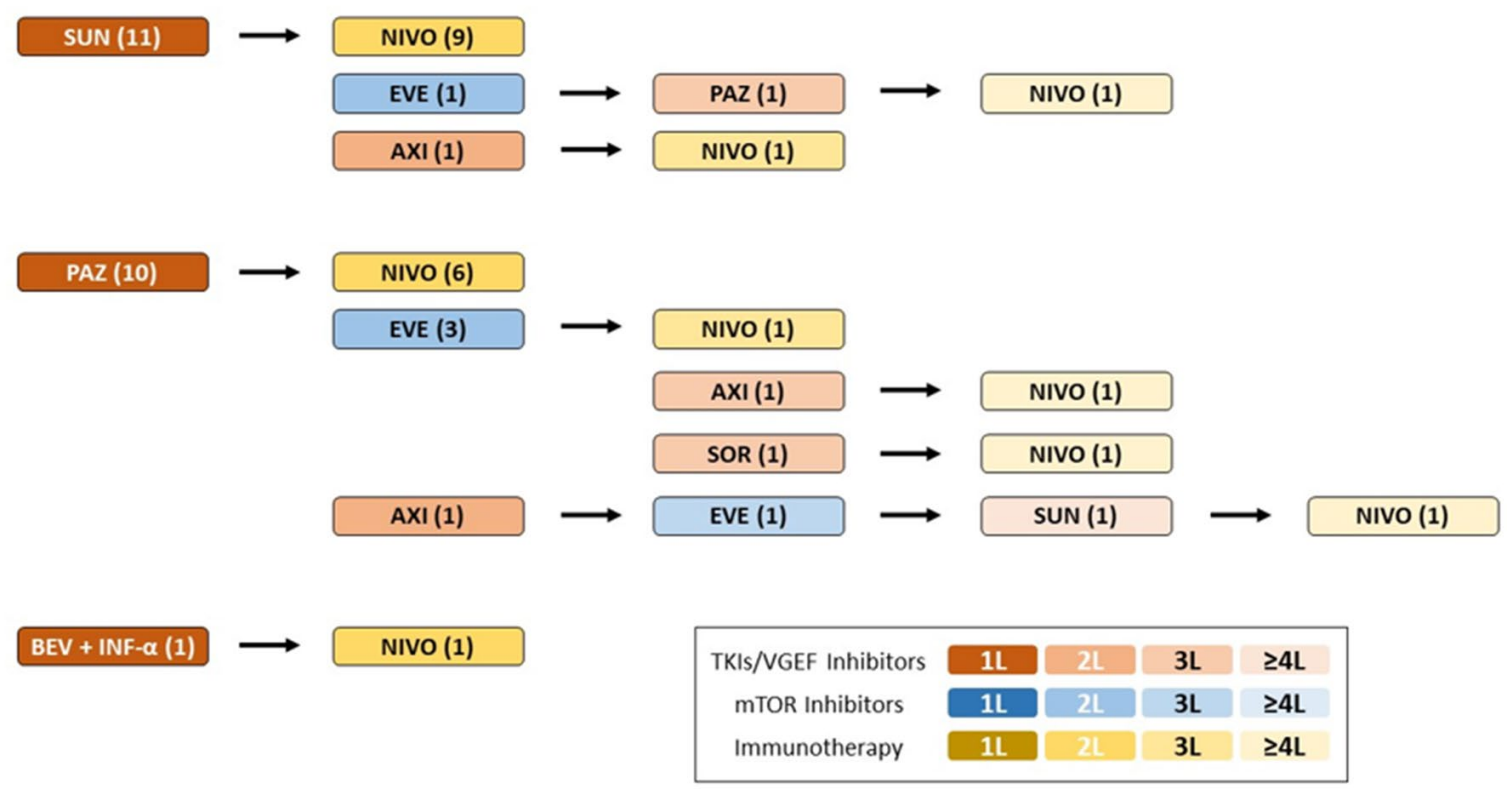
Sequences of systemic therapy in the total population (*n* = 22). *SUN* sunitinib, *PAZ* pazopanib, BEV + INF-α = bevacizumab plus interferon alfa, *NIVO* nivolumab, *EVE* everolimus, *AXI* axitinib, *SOR* sorafenib

This study included 22 (10 women and 12 men) patients with mRCC who received treatment with nivolumab between May 2016 and January 2020. Baseline characteristics are presented in Table 1. The female population was significantly older than the male patients (median age: 70.5 vs. 61.5; *p* = 0.024). The majority (n = 18; 81.8%) of patients had histology of clear cell RCC, three patients had papillary RCC and one individual a chromophobe RCC. According to IMDC criteria, three patients were classified as poor risk, eight counted toward the favorable and nine toward the intermediate risk prognostic group. Two patients could not be assigned to a prognostic group according IMDC criteria, because of lack of data values. Patients had received at least one, but no more than five, prior antiangiogenic cancer therapies. Sixteen patients received nivolumab in second line, two patients in third line, three in fourth line and one patient in fifth line setting (Fig. 1).

**Table 1:** Baseline patient characteristics

	Females	Males	*P* value
*Patients No. (%)*			
Age at therapy start with nivolumab	10	12	
Findings/missing	10/0	12/0	0.024
Mean ± SD/median (IQR)	70.60 ± 9.06/70.5 (54–85)	60.42 ± 10.66/61.50 (43–76)	
*PFS nivolumab in months*			
Findings/missing	10/0	12/0	0.889
Mean ± SD/median (IQR)	11.7 ± 11.31/7.5 (1–34)	11.00 ± 11.85/6 (2–40)	
*OS nivolumab in months*			
Findings/missing	10/0	12/0	0.813
Mean ± SD/median (IQR)	22.2 ± 12.65/20.5 (3–43)	23.42 ± 11.13/20.5 (7–40)	
*Follow-up in months*			
Findings/missing	10/0	12/0	0.171
Mean ± SD/median (IQR)	36.9 ± 18.99/29 (19–76)	51.58 ± 27.67/46 (14–98)	
*Smoking status*			
Findings/missing	10/1	11/1	0.158
yes (%)/no (%)	2 (20)/7 (70)	6 (50)/5 (41.7)	
*ECOG at therapy start with nivolumab*			
Findings/missing	10/0	12/0	0.157
No. ECOG 0 (%)	4 (40)	8 (66.7)	
No. ECOG 1 (%)	5 (50)	4 (33.3)	
No. ECOG 2 (%)	1 (10)	–	
*First-line therapy*			
Findings/missing	9/0	12/0	0.703
No. Sunitinib (%)	6 (60)	5 (41.7)	
No. Pazopanib (%)	3 (30)	7 (58.3)	
No. Bevacizumab (%)	1 (10)	–	
*Therapy-line nivolumab*			
Findings/missing	10/0	12/0	0.361
No. Therapy in 2L (%)	8 (80)	8 (66.7)	
No. Therapy in 3L (%)	1 (10)	1 (8.3)	
No. Therapy in 4L (%)	1 (10)	2 (16.7)	
No. Therapy in 5L (%)	–	1 (8.3)	
Grade			
*Findings/missing*	*9/1*	*12*	*0.830*
G1 (%)	4 (40)	1 (8.3)	
G2 (%)	4 (40)	4 (33.3)	
G3 (%)	1 (10)	4 (33.3)	
G4 (%)	–	3 (25.0)	
*Histology*			
Findings/missing	10/0	12/0	0.064
Clear cell renal carcinoma (%)	10 (100)	8 (66.7)	
Papillary renal cell carcinoma (%)	–	3 (25)	
Chromophobe renal cell carcinoma (%)	–	1 (8.3)	
*Metastasis at therapy start with nivolumab*			
Findings/missing	10/0	12/0	0.976
1 metastasis (%)	1 (10)	3 (25)	
2 metastasis (%)	3 (30)	1 (8.3)	
> 4 metastasis (%)	6 (60)	8 (66.7)	
*IMDC—Score*			
Findings/missing	9/1	11/1	0.679
1	3 (30)	6 (50)	
2	5 (50)	3 (25)	
3	1 (10)	2 (16.7)

An overview of sex-specific response rates is shown in Table 2. The ORR was 22.7% (n = 5) for the total population, 30% (n = 3) in female and 16.7% (n = 2) in male patients. Two female patients achieved CR, one PR and three SD as best overall response. In males, four patients achieved PR and two remained with SD. No significant gender-specific differences were noticed regarding therapy response. Median duration of PFS and OS follow-up after therapy initiation were 7.5 (range: 1–34) and 20.5 (range: 3–43) months in females, as well as 6 (range: 2–40) and 20.5 (7–40) in males.

**Table 2:** Sex-specific response rates

	Females	Males	*P* value
Patients No	10	12	
Therapy response after 12 weeks			
CR, n (%)	–	–	0.516
PR, n (%)	3 (30)	2 (16.7)	
SD, n (%)	3 (30)	4 (33.3)	
PD, n (%)	4 (40)	6 (50)	
ORR, n (%)	3 (30)	2 (16.7)	0.481
Best overall response, n (%)			
CR, n (%)	2 (20)	–	0.564
PR, n (%)	1 (10)	4 (33.3)	
SD, n (%)	3 (30)	2 (16.7)	
PD, n (%)	4 (40)	6 (50)	

### Sex-specific hormone characteristics

The hormone values for men and women at baseline, at landmark of 6/8 weeks and 12 weeks are presented in Table 3. The median LH/FSH ratio at baseline for women and men was 0.33 (range: 0.06–1.57) and 0.58 (0.28–1.03), 0.36 (range: 0.09–0.67) and 0.685 (range: 0.27–1.03) at 6/8 weeks, 0.4 (range: 0.18–0.76) and 0.72 (range: 0.21–1.34) at landmark of 12 weeks. The median E2 value for women and men at baseline was 13 ng/mL (range: 13–25 ng/mL) and 21 ng/mL (range: 13–33 ng/mL), 13 ng/mL (range: 13–25 ng/mL) and 30.5 (range: 13–39) at 6/8 weeks, 13 ng/mL (range: 13–31) and 29 (range: 15–42) at landmark of 12 weeks.

**Table 3:** Sex-specific hormone characteristics

	Females	Males
Patients No	10	12
LH—week 0 in U/L		
Findings/missing	9/1	12/0
Mean ± SD/median (IQR)	29.23 ± 23.71/23.0 (0.4–60.5)	7.08 ± 3.32/6.15 (3.8–14.9)
LH—week 6/8 in U/L		
Findings/missing	10/0	12/0
Mean ± SD/median (IQR)	27.95 ± 23.41/21.4 (0.3–65.0)	6.57 ± 3.47/5.75 (3.3–16)
LH—week 12 in U/L		
Findings/missing	10/0	12/0
Mean ± SD/median (IQR)	32.13 ± 21.10/24.4 (13.1–76.7)	7.55 ± 5.7/5.2 (4.0–21.3)
FSH—week 0 in U/L		
Findings/missing	9/1	11/1
Mean ± SD/median (IQR)	75.90 ± 50.12/69.4 (4.8–170.0)	13.92 ± 6.97/10.9 (6.4–29.3)
FSH—week 6/8 in U/L		
Findings/missing	10/0	12/0
Mean ± SD/median (IQR)	67.38 ± 45.63/56.6 (3.3–170.0)	11.07 ± 6.12/9.3 (4.2–25.4)
FSH—week 12 in U/L		
Findings/missing	10/0	12/0
Mean ± SD/median (IQR)	73.59 ± 35.62/67.95 (21.0–151.0)	10.93 ± 6.24/8.7 (4.1–24.8)
LH/FSH ratio—week 0		
Findings/missing	9/1	11/1
Mean ± SD/median (IQR)	0.32 ± 0.17/0.33 (0.06–0.57)	0.58 ± 0.24/0.58 (0.28–0.89)
LH/FSH ratio—week 6/8		
Findings/missing	10/0	12/0
Mean ± SD/median (IQR)	0.36 ± 0.18/ 0.36 (0.09–0.67)	0.68 ± 0.28/0.69 (0.27–1.03)
LH/FSH ratio—week 12		
Findings/missing	10/0	12/0
Mean ± SD/median (IQR)	0.44 ± 0.16/0.4 (0.18–0.76)	0.77 ± 0.34/0.72 (0.21–1.34)
E2—week 0 in ng/mL		
Findings/missing	9/1	12/0
Mean ± SD/median (IQR)	17.00 ± 5.10/13.0 (13–25)	22.25 ± 7.20/21.00 (13–33)
E2—week 6/8 in ng/mL		
Findings/missing	10/0	12/0
Mean ± SD/median (IQR)	14.80 ± 4.05/13.00 (13–25)	29.00 ± 6.84/30.50 (13–39)
E2—week 12 in ng/mL		
Findings/missing	10/0	12/0
Mean ± SD/median (IQR)	16.60 ± 6.45/13.00 (13–31)	28.58 ± 7.01/29 (15–42)
Testosterone—week 0 in µg/mL		
Findings/missing	9/1	12/0
Mean ± SD/median (IQR)	0.17 ± 0.08/0.14 (0.10–0.33)	3.73 ± 1.58/3.56 (1.48–7.61)
Testosterone—week 6/8 in µg/mL		
Findings/missing	10/0	12/0
Mean ± SD/median (IQR)	0.15 ± 0.5/0.14 (0.10–0.24)	3.90 ± 1.18/4.08 (2.47–6.05)
Testosterone—week 12 in µg/mL		
Findings/missing	10/0	12/0
Mean ± SD/median (IQR)	0.15 ± 0.06/0.13 (0.10–0.25)	3.63 ± 1.18/3.58 (1.73–6.09)
Prolactin—week 0 in µg/mL		
Findings/missing	10/0	10/2
Mean ± SD/median (IQR)	14.20 ± 18.12/9.5 (6.00–65.90)	11.38 ± 9.72/8.40 (2.20 – 35.30)
Prolactin—week 6/8 in µg/mL		
Findings/missing	10/0	12/0
Mean ± SD/median (IQR)	16.69 ± 22.30/9.95 (5.10–79.30)	11.85 ± 7.64/10.05 (3.50–30.60)
Prolactin—week 12 in µg/mL		
Findings/missing	10/0	12/0
Mean ± SD/median (IQR)	13.25 ± 12.00/9.70 (6.80–45.30)	10.78 ± 7.15/8.70 (2.40–25.80)

We did not detect any significant associations between hormone levels/hormone ratios and descriptive patient characteristics neither at baseline nor at 6/8 or 12 weeks.

### Sex-specific changes of FSH, E2 levels and LH/FSH ratios during immunotherapy with nivolumab

**Fig. 2:**
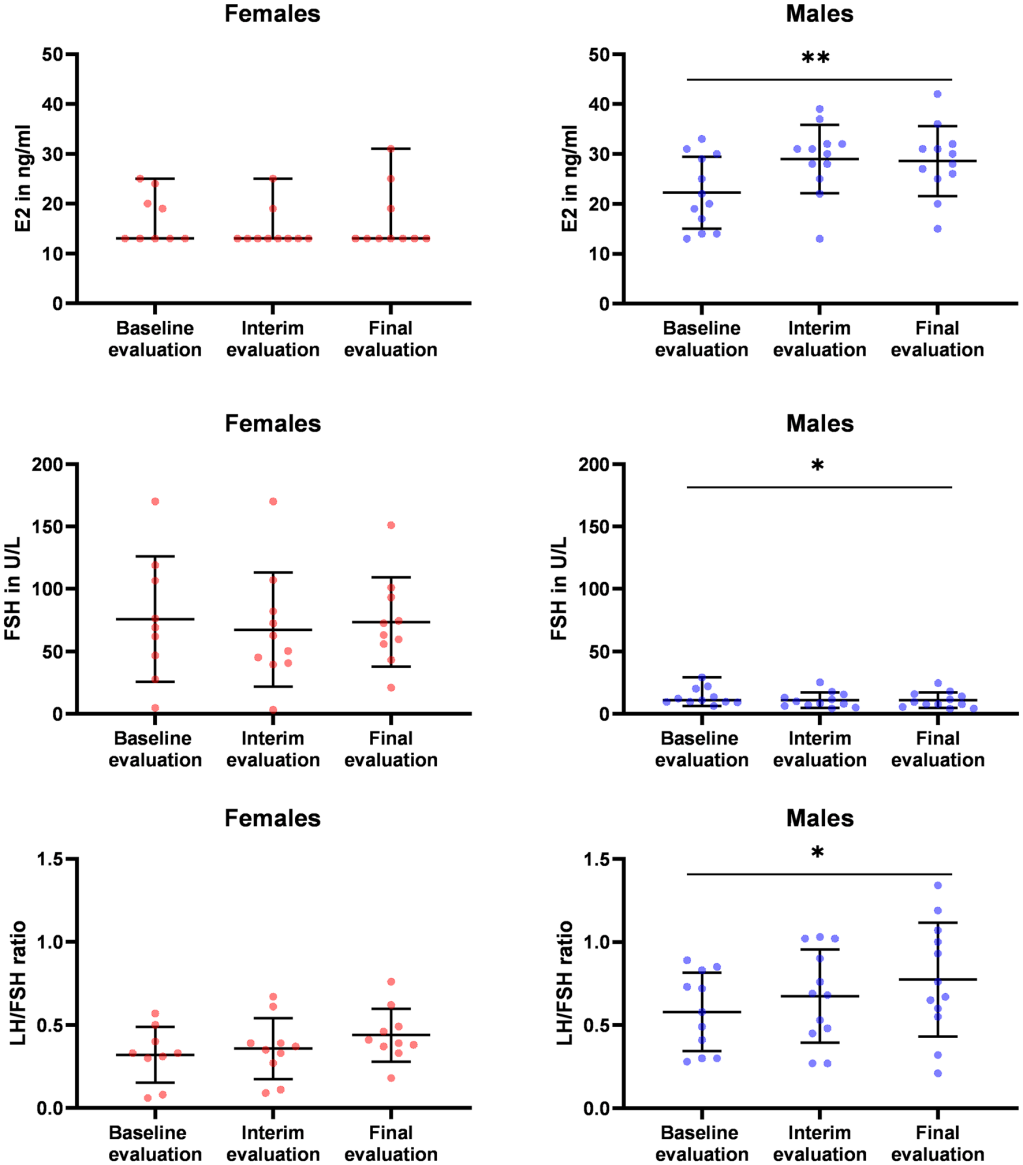
Dynamic changes of **a** 17-ß-estradiol (E2); **b** FSH and **c** LH/FSH ratio during nivolumab therapy stratified by sex. Evaluations were performed at week 0 (baseline evaluation), 6/8 week (interim evaluation) and week 12 (final evaluation). Changes are tested for significance between treatment begin (week 0) and week 12. The bar graph is showing mean ± SD for all hormone parameters with the exception of E2 in females. Here, the data are not normally distributed, so median and range were used. * *p* < 0.05; ** *p* < 0.01; *** *p* < 0.001

During the period of treatment begin (week 0) and week 12, a significant increase in E2 levels in male patients could be observed (*p* = 0.006) (Fig. 2). In contrast, no significant changes in E2 levels were observed in female patients. We could additionally detect a significant increase in the LH/FSH ratio from baseline to week 12 in male patients (*p* = 0.013; borderline after Bonferroni correction) but not in female patients (Fig. 2). Likewise, FSH levels showed a significant decrease from baseline to week 12 in males (*p* = 0.011) but not in female patients (Fig. 2). No significant changes of LH, testosterone and prolactin levels were observed in the female as well as in the male population (supplementary Fig. 1).

### Increased LH/FSH ratio at interim analysis was significantly associated with immunotherapy response and survival

**Fig. 3:**
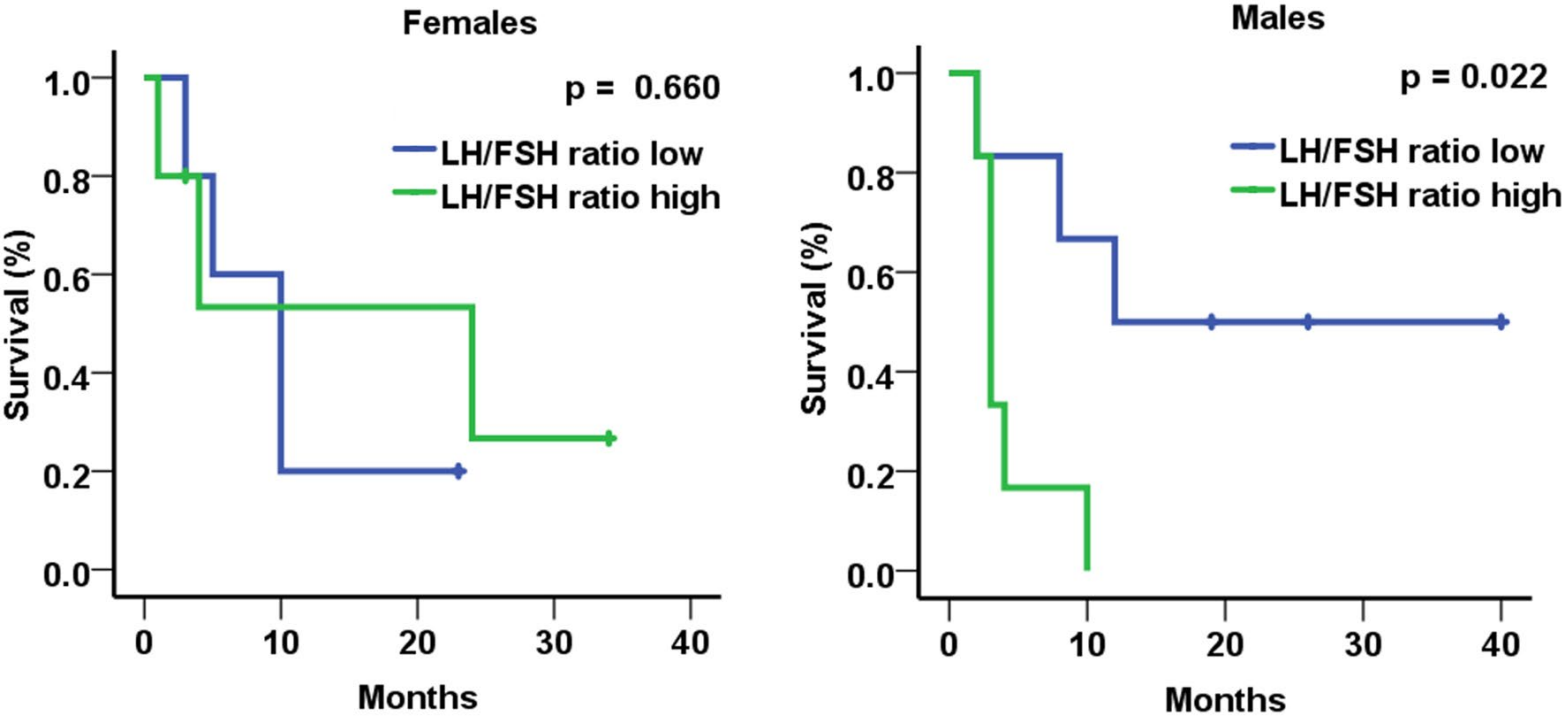
PFS according to LH/FSH ratio at 6/8 weeks landmark (low vs. high, dichotomized at the median of 0.69 for male population and at the median of 0.36 for female population. The bar graph is showing mean ± SD

**Fig. 4:**
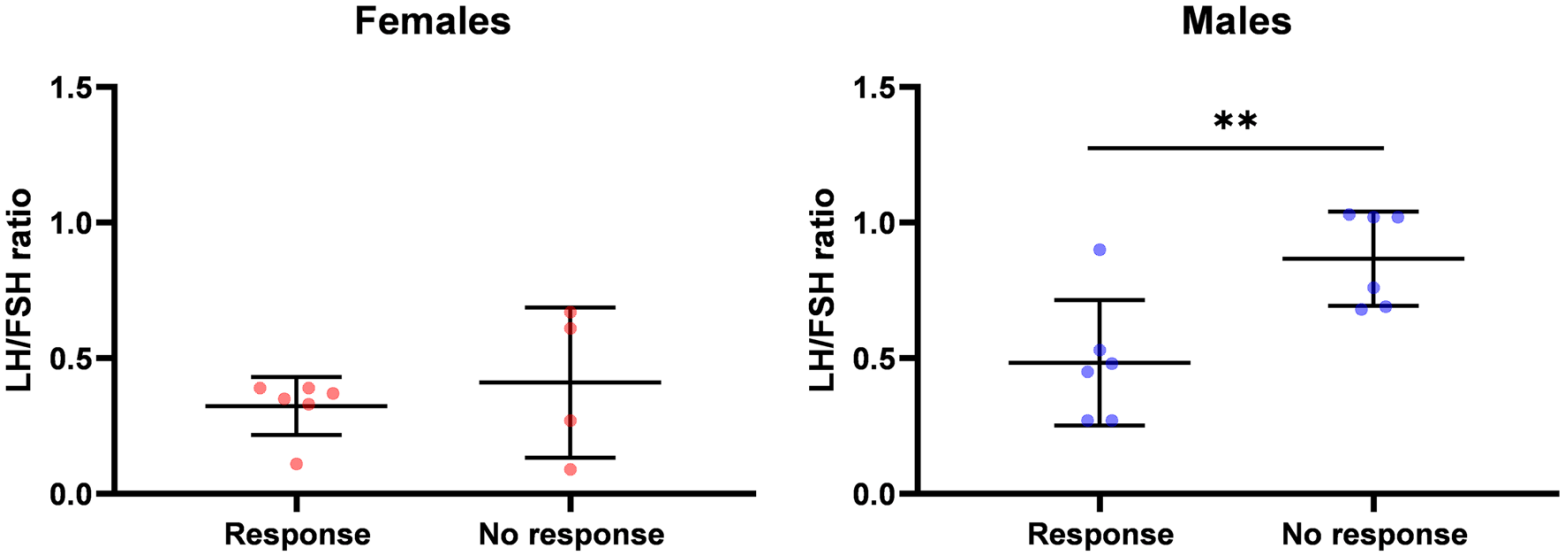
LH/FSH ratio at landmark of 6/8 weeks is associated with therapy response for the male but not for the female population. Therapy response was defined as SD, PR, CR by RECIST in monitoring CT after 12 weeks of treatment with nivolumab. The bar graph is showing mean ± SD. * *p* < 0.05; ** *p* < 0.01; *** *p* < 0.001

Finally, we analyzed potential sex-specific associations between sex hormone levels either assessed at baseline or at 6/8 and 12 weeks with response to nivolumab (SD, PR, CR) or outcomes (PFS, OS). At interim analysis, the association between LH/FSH ratio and PFS was apparent in male but not in female patients (Fig. 3). Moreover, the LH/FSH ratio at 6/8 weeks also correlated with response to therapy in male patients (Fig. 4). LH/FSH ratios did not correlate with OS, neither in male nor in female patients. Finally, no other endocrine parameters correlated with response, PFS or OS in women or men (supplementary Figs. 2–14**)**.

**Fig. 5:**
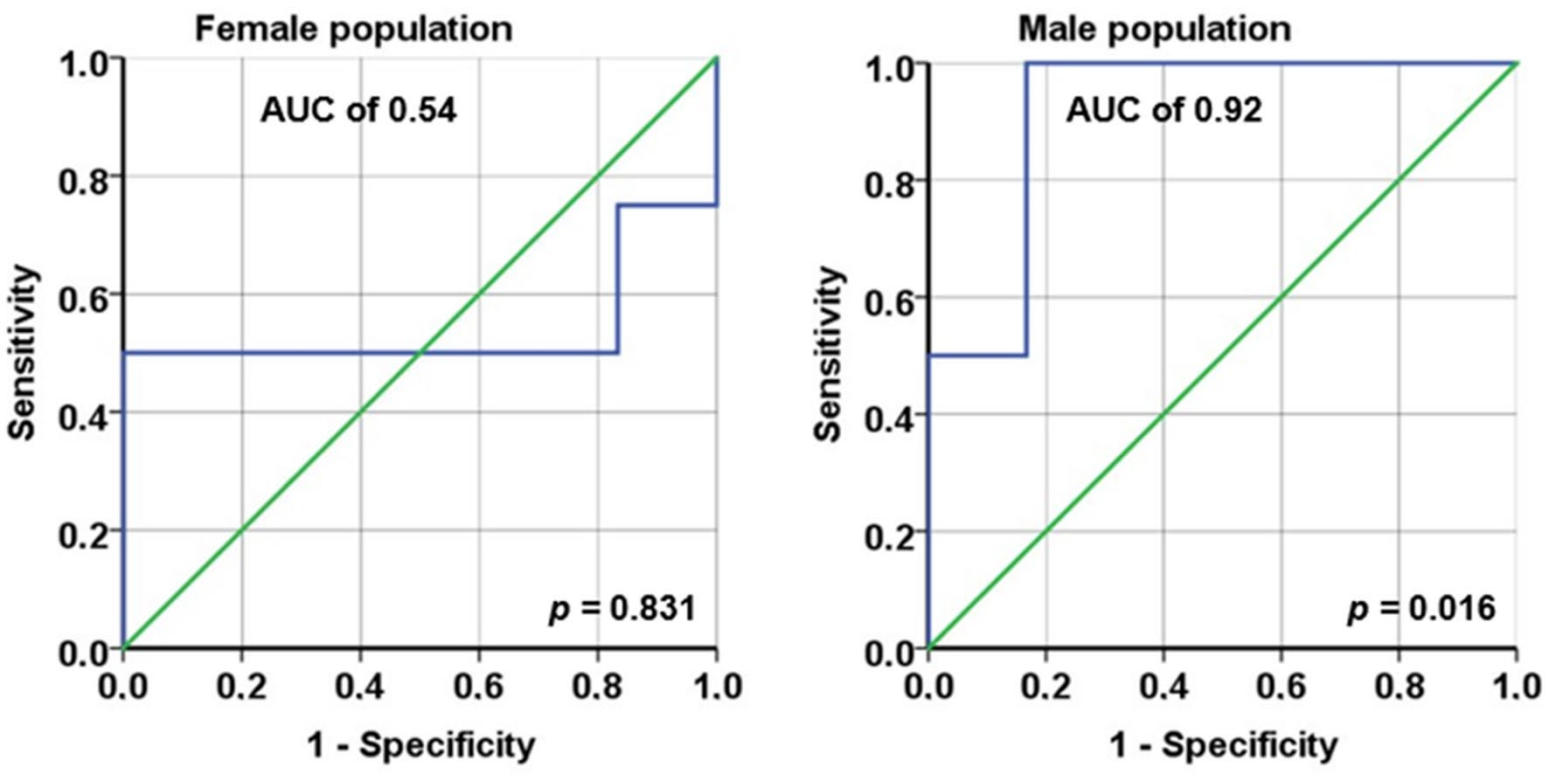
Sex-specific ROC curves for LH/FSH ratio (at 6/8 weeks) to predict progression

A ROC analysis of LH/FSH ratio at interim analysis showed an AUC of 0.92 (95% CI. 0.742–1.000; *p* = 0.016) to predict progression for males. The best cutoff calculated by highest Youden Index was at 0.605 for men. Sensitivity, specificity, PPV and NPV were shown to be 100%, 83%, 74% and 100%, respectively. ROC analysis for the female population revealed no significant results (AUC = 0.54; 95% CI: 0.08–1.00; *p* = 0.831) (Fig. 5).

## Discussion

Sex differences in immune responses, including antitumor responses have been reported in the past [34, 35]. In meta-analyses, immune checkpoint inhibitors as single agents appear to be more effective in male cancer patients compared with female patients [23, 24, 36]. This controversially discussed data do not necessarily apply to RCC [37]. Potential effects of checkpoint inhibition on sex hormones have not yet been fully explored, leaving it more difficult to interpret current data.

In present work, we report on changes in sex hormones in mRCC patients undergoing nivolumab therapy. We observed a significant increase in E2 levels in male but not in female patients between treatment start and the last follow-up at week 12. In addition, we could detect a significant increase in the LH/FSH ratio from baseline to week 12 in male patients but not in female patients. Our findings indicate that the increase in the LH/FSH ratio is most likely due to a decrease in FSH levels. At interim analysis at 6/8 weeks, an association between LH/FSH ratio and PFS was apparent in male but not in female patients. Moreover, the LH/FSH ratio at 6/8 weeks also correlated with response to therapy in male patients, yet LH/FSH ratios did not correlate with OS, neither in male nor in female patients.

The nivolumab-induced increase in E2 levels in male patients is of interest, because in vitro data as well as preclinical and clinical evidence suggest a role for E2 in the upregulation of PD-1 and PD-L1 expression [29, 38, 39]. The PD-L1 pathway has also been implicated in feto-maternal tolerance during pregnancy both in animal models [40] and in humans [29]. Female hormones such as E2 are supposed to maintain feto-maternal tolerance by enhancing PD-1 expression in several types of antigen-presenting cells and in regulatory T cells [29], thus preventing an immune-mediated fetal rejection. Similarly, E2-driven tumor tolerance may limit the efficacy of nivolumab-based immunotherapy. In addition to positive and negative E2 effects on immune cells [25, 26, 30], E2 has also been shown to directly promote RCC growth in a mouse model [41]. Moreover, women with five or more births had a two-fold increase in mRCC risk when compared to those with one or two births [42]. In animal melanoma models, PD-1 inhibition using nivolumab has been shown to increase IL-6 production by macrophages in the tumor microenvironment. In melanoma patients, increased systemic levels of IL-6 were associated with poor clinical response [43]. IL-6 can stimulate E2 synthesis [44], then again E2 further upregulates PD-1 and PD-L1 expression [29, 38, 39], generating a feedforward immunosuppressive mechanism. In estrogen receptor α (ERα), positive endometrial and breast cancer cell lines E2 can increase PD-L1 protein expression via the phosphoinositide 3 kinase (PI3K)/Akt pathway and post-transcriptional PD-L1 mRNA stabilization [45].

Extragonadal E2 production has been shown in non-small cell lung cancer (NSCLC). E2 concentration in NSCLC tissue was 2.2 fold higher than basal levels in non-neoplastic lung tissues [46]. Increased E2 levels were positively correlated with aromatase expression in tumor tissues, suggesting local E2 production [46]. High aromatase expression has also been demonstrated to be a poor predictor of survival in both sexes in early stage NSCLC patients, especially in women ≥ 65 years [47]. Additionally, the combination of ERβ and aromatase expression in NSCLC was a stronger predictor of poor survival than ERβ alone in women and men [47].

In our male patients, we observed an increase in E2 during nivolumab therapy. In contrast, testosterone remained unaffected. This suggests extragonadal E2 synthesis in RCC patients similar to NSCLC patients [47]. LH and FSH are members of the gonadotropin family of pituitary hormones that are secreted by the anterior pituitary gland. FSH can stimulate gonadal E2 production and conversely increasing E2 levels can result in a negative feedback on pituitary to attenuate FSH secretion [48, 49]. In line with this, a reduction in FSH during immunotherapy could be detected in our male patients. However, we did not find any association between E2 levels and therapy response, which would indicate its immunosuppressive effects. Instead, LH/FSH ratios were negatively associated with therapy response and PFS in males at interim analysis. Why the observed effect was undetectable at the last follow-up is currently unclear. Notably, the role of LH/FSH ratio has not yet been investigated in RCC. Increased E2 production may in fact result from nivolumab-mediated PD-1 blockade, representing an attempt to enhance E2-driven PD-1 expression in order to maintain immunological tolerance. Concomitantly, E2-mediated attenuation of FSH secretion through negative feedback on pituitary may cause the increase in LH/FSH ratios. The correlation of high LH/FSH ratio with poor PFS and therapy failure at interim analysis may thus reflect pro-tumor effects of nivolumab-induced E2 regulation. On the other hand, during prolonged treatment, E2-driven PD-1 expression would permanently offer additional nivolumab targets, a beneficial E2 effect that may eliminate the correlation between LH/FSH ratio and PFS/therapy failure at the final analysis.

Beneficial effects of E2 have also been described. A population-based study recently reported that female sex was a significant predictor of poor renal cancer-specific mortality for advanced disease, however, only in postmenopausal period [50]. In addition, E2 has also been shown to inhibit RCC growth in vitro via an estrogen receptor-ß (ER-ß) dependent pathway [51]. Finally, hormonal agents such as medroxyprogesterone were initially shown to be active in metastatic RCC, although the response rates reported in various studies were modest, ranging from 7 to 25% [52, 53]. RCC incidence seems to increase in females after hysterectomy, suggesting that a decrease in E2 after hysterectomy may be one of the causes of this increased risk [54]. However, our female patients demonstrated neither E2 upregulation nor feedback inhibition of FSH secretion in response to nivolumab. All of them were postmenopausal, none of them receiving any type of hormone replacement therapy. Since RCC usually occurs between the 6^th^ and 7^th^ decade of life, age becomes a surrogate for hormone status and female RCC patients will most commonly be postmenopausal.

Our study has certain limitations. The most restrictive fact is our small population number and the short follow-up after therapy start. Especially for OS (median follow-up of only 23.8 months), a follow-up completeness is essential for reliable outcome assessments [55]. In such a small population, the heterogeneous group with women still being underrepresented compared with men, could have a significant influence on study results with limited statistical power, as ROC analysis cannot be considered very reliable. Moreover, we could include only postmenopausal patients. Further, our patient collective received diverse pre-treatments. Only sixteen patients were treated with nivolumab in second line and the possible effect on sex hormone changes by the previous multiple treatment modalities in other six patients remains unclear. Thus, our results are only hypothesis-generating and no definitive conclusions can be drawn yet.

In summary, we present first data on sex hormone modulation during immune checkpoint inhibition in patients with mRCC. An increase in E2, LH/FSH ratio and a decrease in FSH in males but not in females could be observed. LH/FSH ratio at interim analysis correlated with PFS and response rates. Our findings suggest further investigation of sex hormones and its association with efficacy of cancer immunotherapy to elucidate the complete mechanisms of such findings. Monitoring of sex hormones to evaluate these parameters as possible prognostic markers and the analysis of the interaction between sex hormone signaling and antitumor immunity during immune checkpoint inhibition should be the focus of larger clinical trials.

### Supplementary Information

Below is the link to the electronic supplementary material.

Supplementary file1 (PDF 1,547 kb)

## Abbreviations

AEs: Adverse events
ANC: Absolute neutrophil count
AUC: Area under the curve
CT: Computed tomography
CR: Complete remission
DCs: Dendritic cells
E2: 17-ß-estradiol
ER-α: Estrogen receptor-α
ER-ß: Estrogen receptor-ß
FSH: Follicle stimulating hormone
IFN-α: Interferon alpha
IL-2: Interleukin-2
IMDC: International Metastatic RCC Database Consortium
IQR: Interquartile ranges
IV: Intravenous
KPS: Karnofsky Performance Status
LH: Luteinizing hormone
mRCC: Metastatic renal cell carcinoma
mTOR: Mammalian target of rapamycin
OR: Objective response rate
OS: Overall survival
PD-1: Programmed cell death-1
PD-L1: Programmed cell death ligand 1
PFS: Progression-free survival
PR: Partial remission
RCC: Renal cell carcinoma
ROC: Receiver operating characteristic
SD: Stable disease
TILs: Tumor-infiltrating lymphocytes
TKIs: Tyrosine kinase inhibitors
