# Supplementary material for: Sex-specific hormone changes during immunotherapy and its influence on survival in metastatic renal cell carcinoma
Source: Cancer Immunol Immunother. 2021 Feb 28;70(10):2805–17. doi: 10.1007/s00262-021-02882-y (PMC8423679; doi:10.1007/s00262-021-02882-y)
Supplement: Supplementary file 1 — Supplementary file1 (PDF 1,547 kb) [file 262_2021_2882_MOESM1_ESM.pdf]

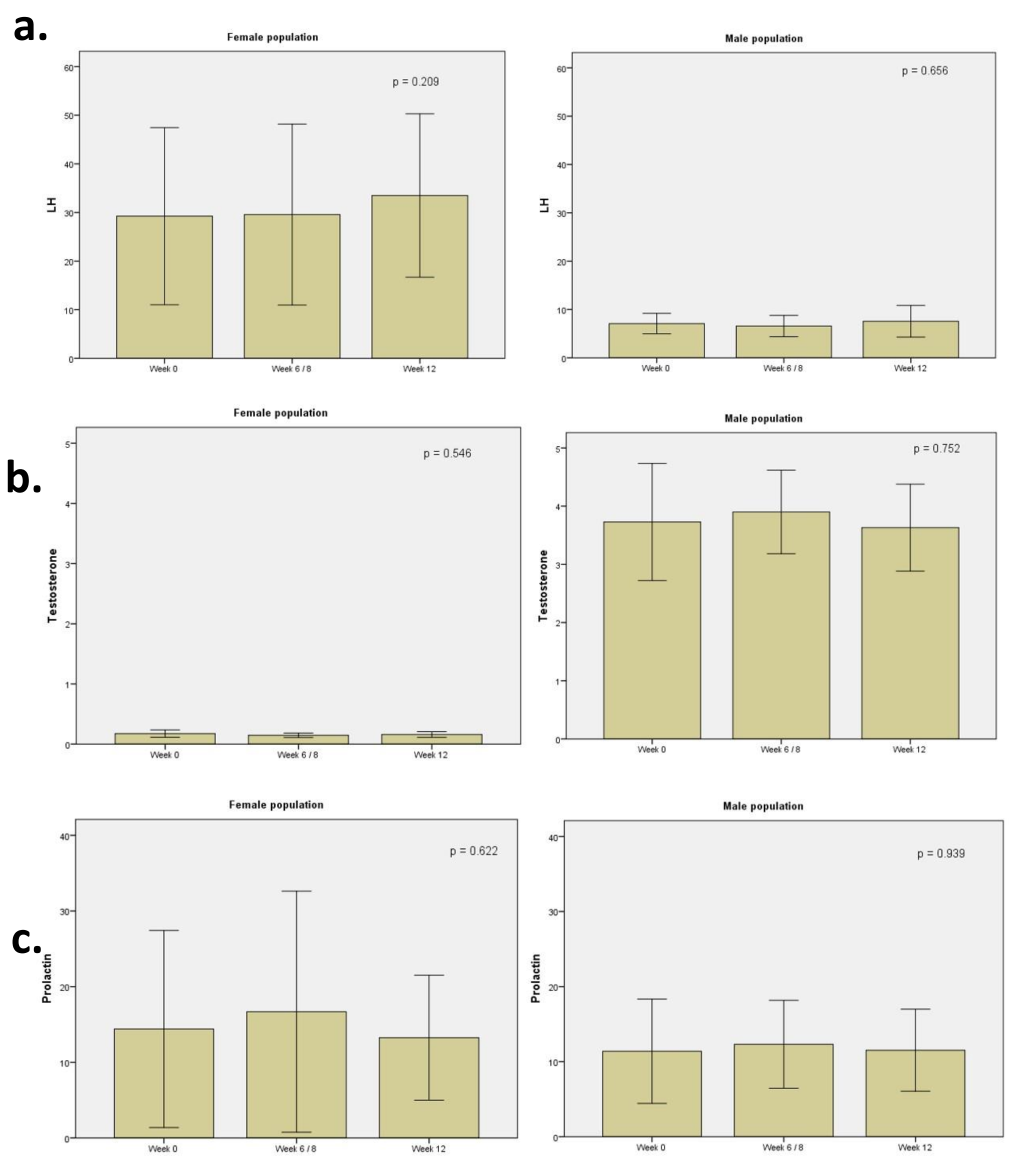

Supplementary Figure 1:
(a) LH in U/L; (b) Testosterone in µg/ml and (c) Prolactin in µg/ml during nivolumab therapy stratified by gender. Evaluations were performed at week 0 (baseline evaluation), 6 / 8 week (interim evaluation) and week 12 (final evaluation).

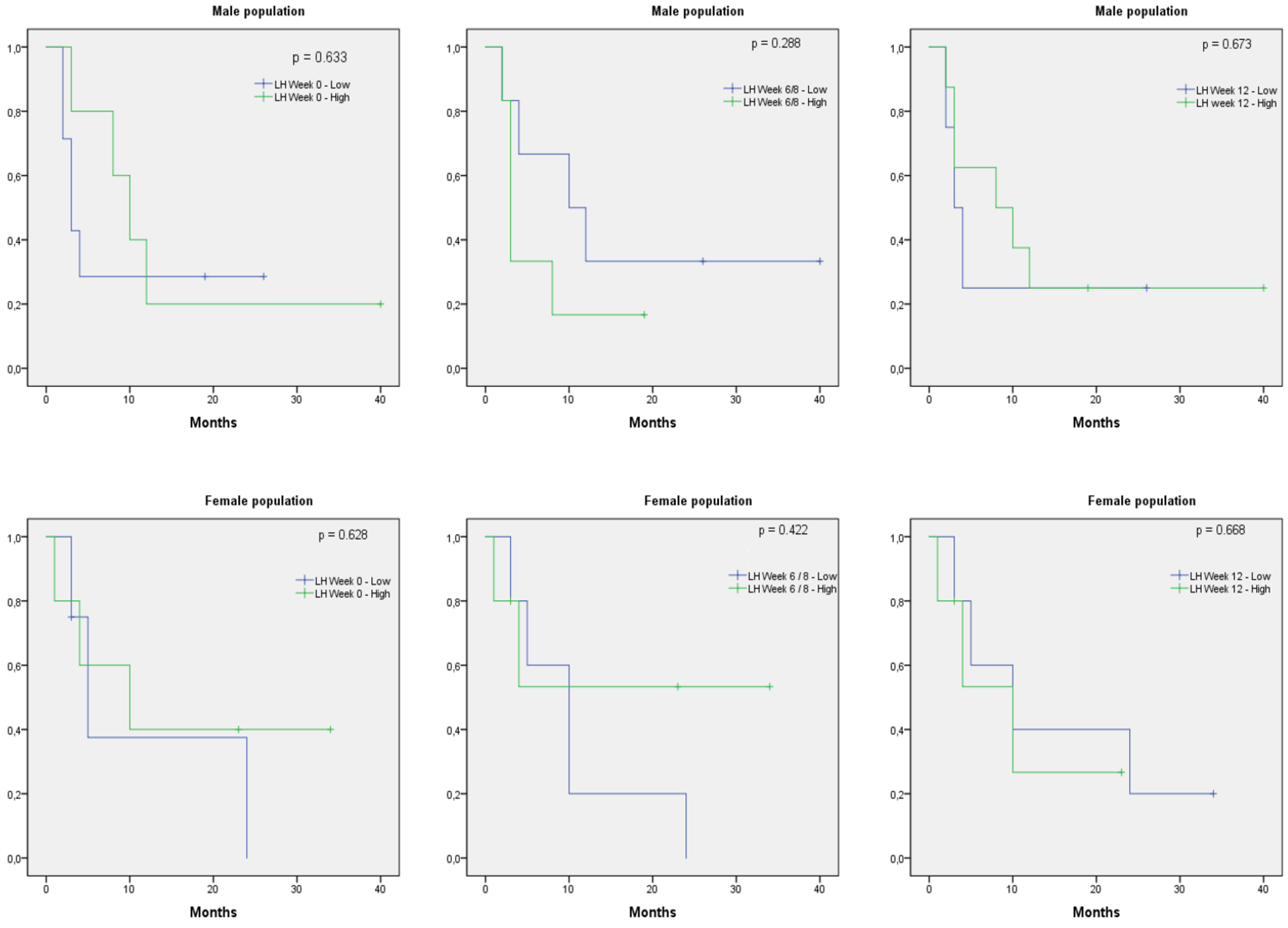

Supplementary Figure 2:
PFS according to LH, dichotomized in low vs. high at the median of 6.15, 5.75 and 5.2 U/L for male population as well as at the median of 23.0, 21.4 and 24.4 U/L for female population at the three landmark evaluations performed at week 0 (baseline evaluation) 6/8 week (interim evaluation) and week 12 (final evaluation). ~~*$p < 0.05$; **$p < 0.01$; ***$p < 0.001$~~

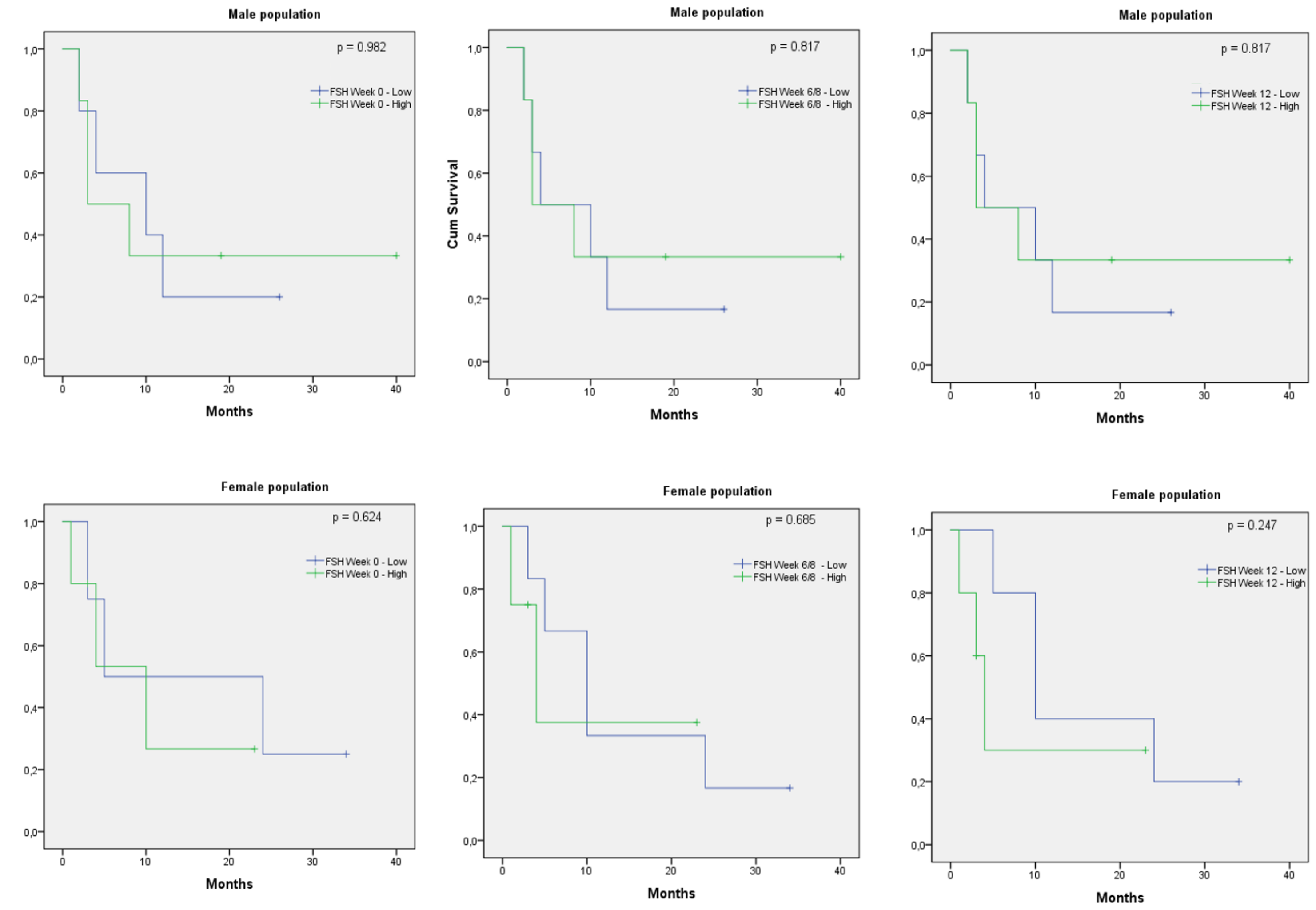

Supplementary Figure 3:

PFS according to FSH, dichotomized in low vs. high at the median of 10.9, 9.3 and 8.7 U/L for male population as well as at the median of 69.4, 56.6 and 67.95 U/L for female population at the three landmark evaluations performed at week 0 (baseline evaluation) 6/8 week (interim evaluation) and week 12 (final evaluation). ~~*$p < 0.05$; **$p < 0.01$; ***$p < 0.001$~~

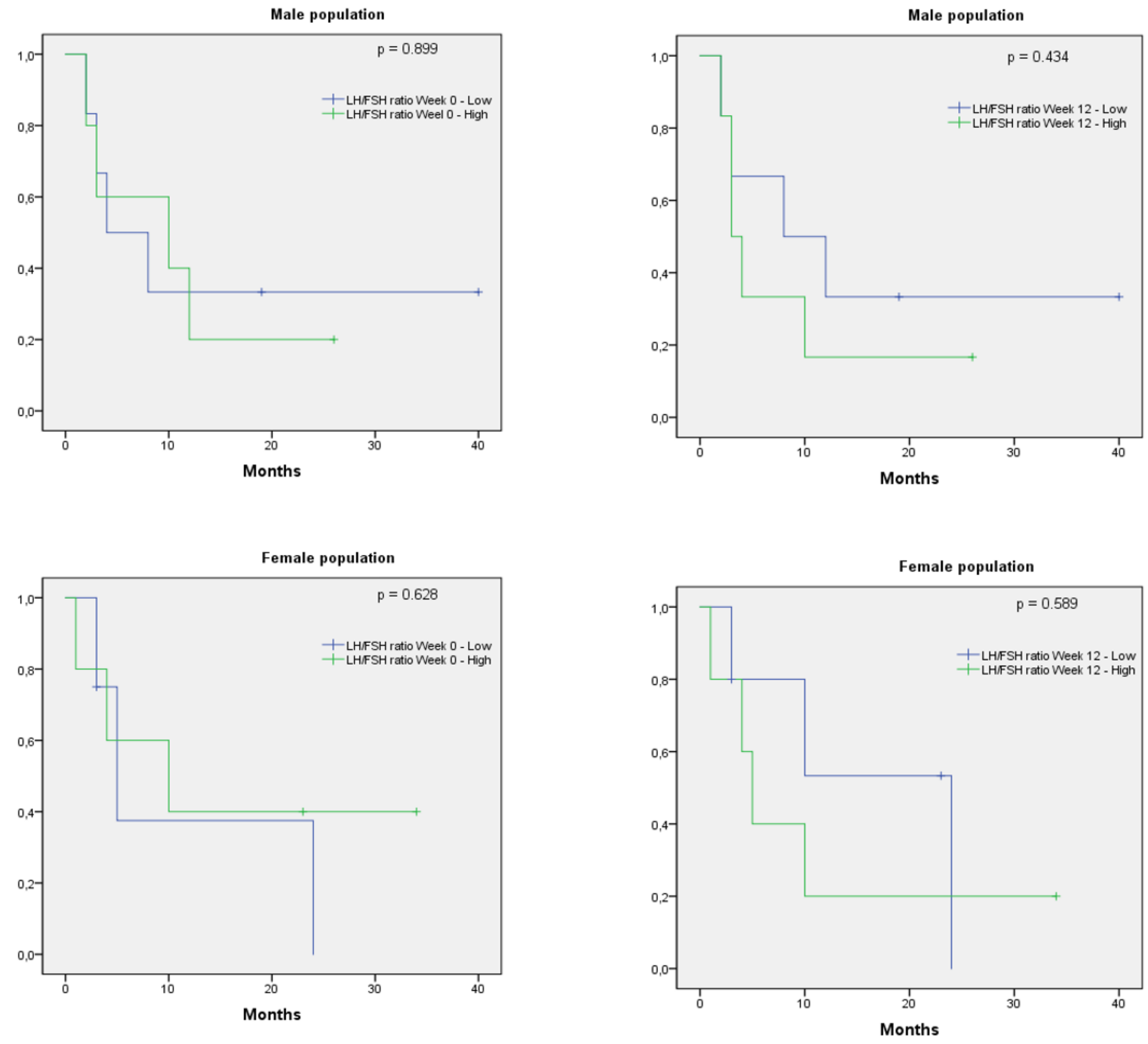

Supplementary Figure 4:
PFS according to LH/FSH ratio, dichotomized in low vs. high at the median of 0.58 and 0.72 for male population as well as at the median of 0.33 and 0.4 for female population at the landmark evaluations performed at week 0 (baseline evaluation) and week 12 (final evaluation). ~~*$p < 0.05$; **$p < 0.01$; ***$p < 0.001$~~

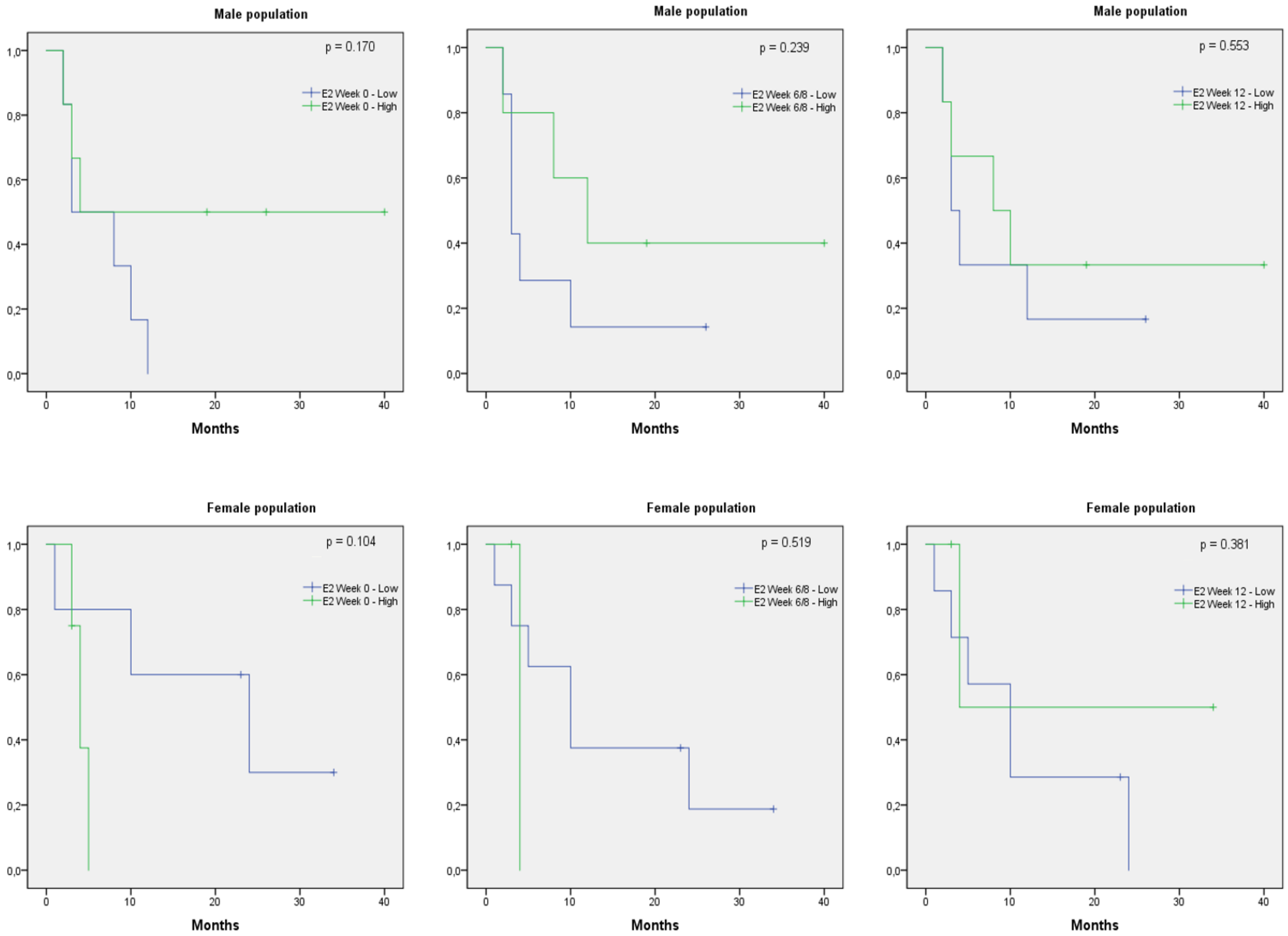

Supplementary Figure 5:
PFS according to E2, dichotomized in low vs. high at the median of 21.0, 30.5 and 29 ng/ml for male population as well as at the median of 13, 13, and 13 ng/ml for female population at the three landmark evaluations performed at week 0 (baseline evaluation) 6/8 week (interim evaluation) and week 12 (final evaluation). ~~*$p < 0.05$; **$p < 0.01$; ***$p < 0.001$~~

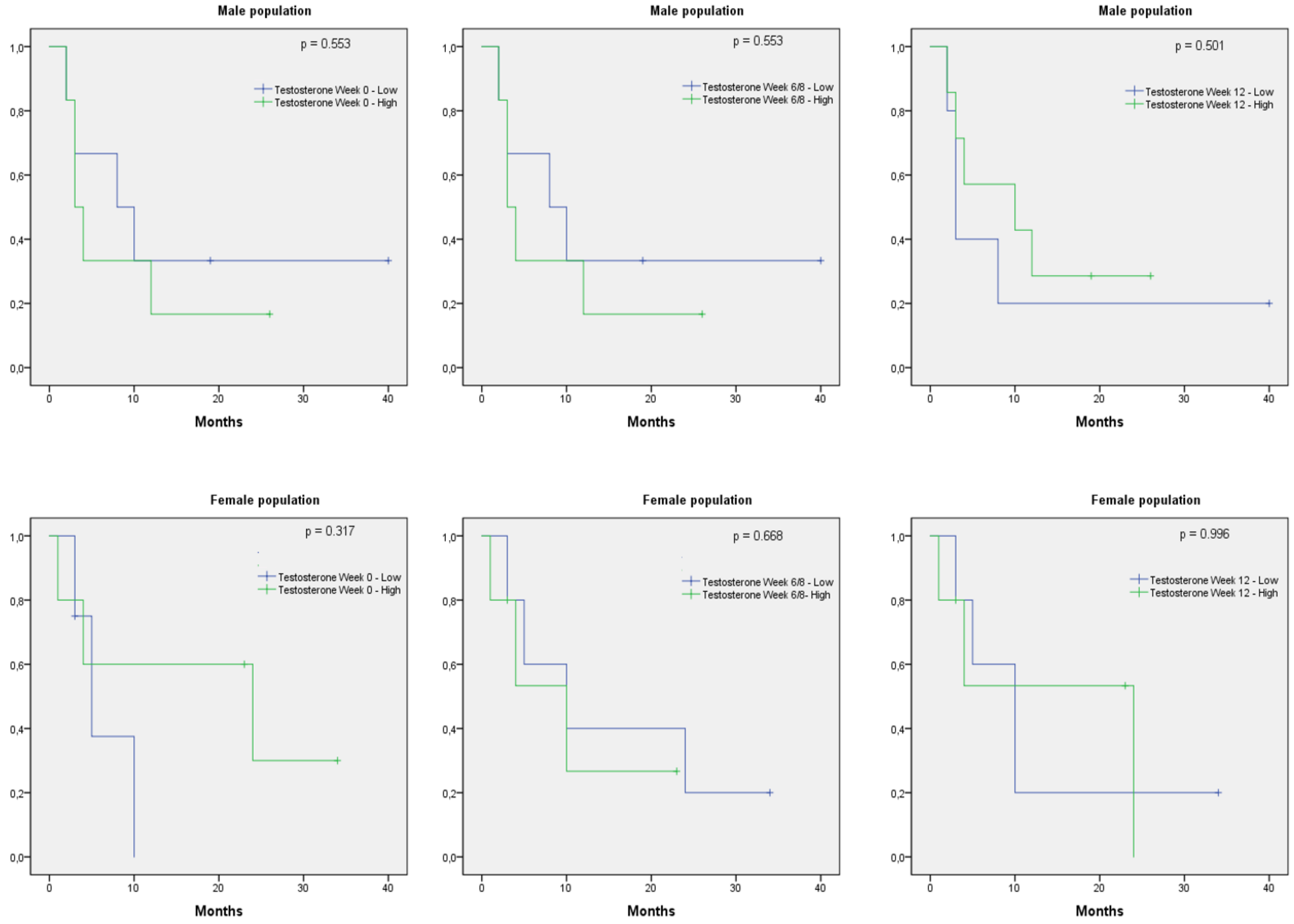

Supplementary Figure 6:
PFS according to Testosterone, dichotomized in low vs. high at the median of 3.56, 4.08 and 3.58 µg/ml for male population as well as at the median of 0.14, 0.14 and 0.13 µg/ml for female population at the three landmark evaluations performed at week 0 (baseline evaluation) 6/8 week (interim evaluation) and week 12 (final evaluation). ~~*$p < 0.05$; **$p < 0.01$; ***$p < 0.001$~~

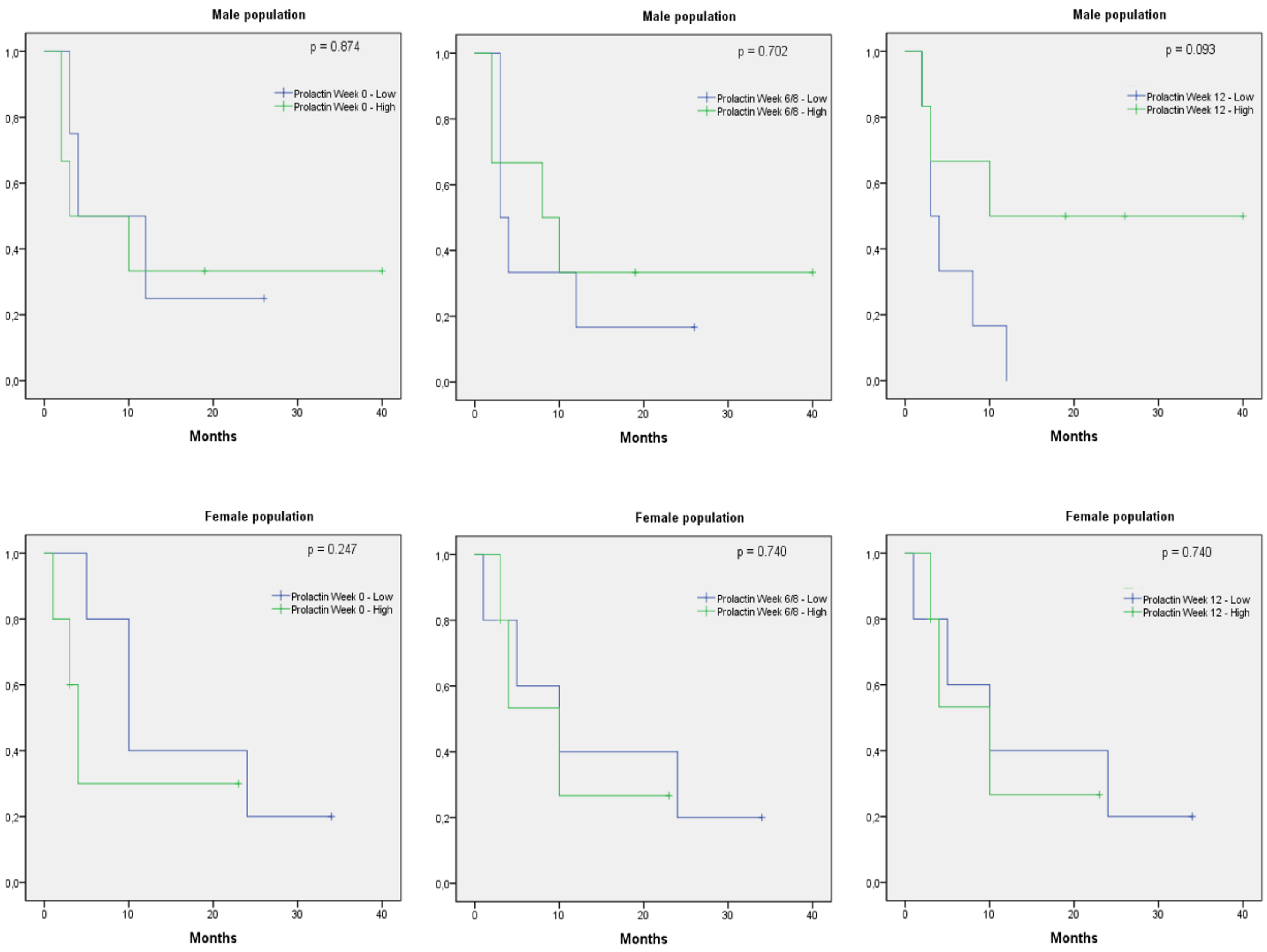

Supplementary Figure 7:
PFS according to Prolactin, dichotomized in low vs. high at the median of 8.4, 10.05 and 8.7 µg/ml for male population as well as at the median of 9.5, 9.95 and 9.7 µg/ml for female population at the three landmark evaluations performed at week 0 (baseline evaluation) 6/8 week (interim evaluation) and week 12 (final evaluation). ~~*$p < 0.05$; **$p < 0.01$; ***$p < 0.001$~~

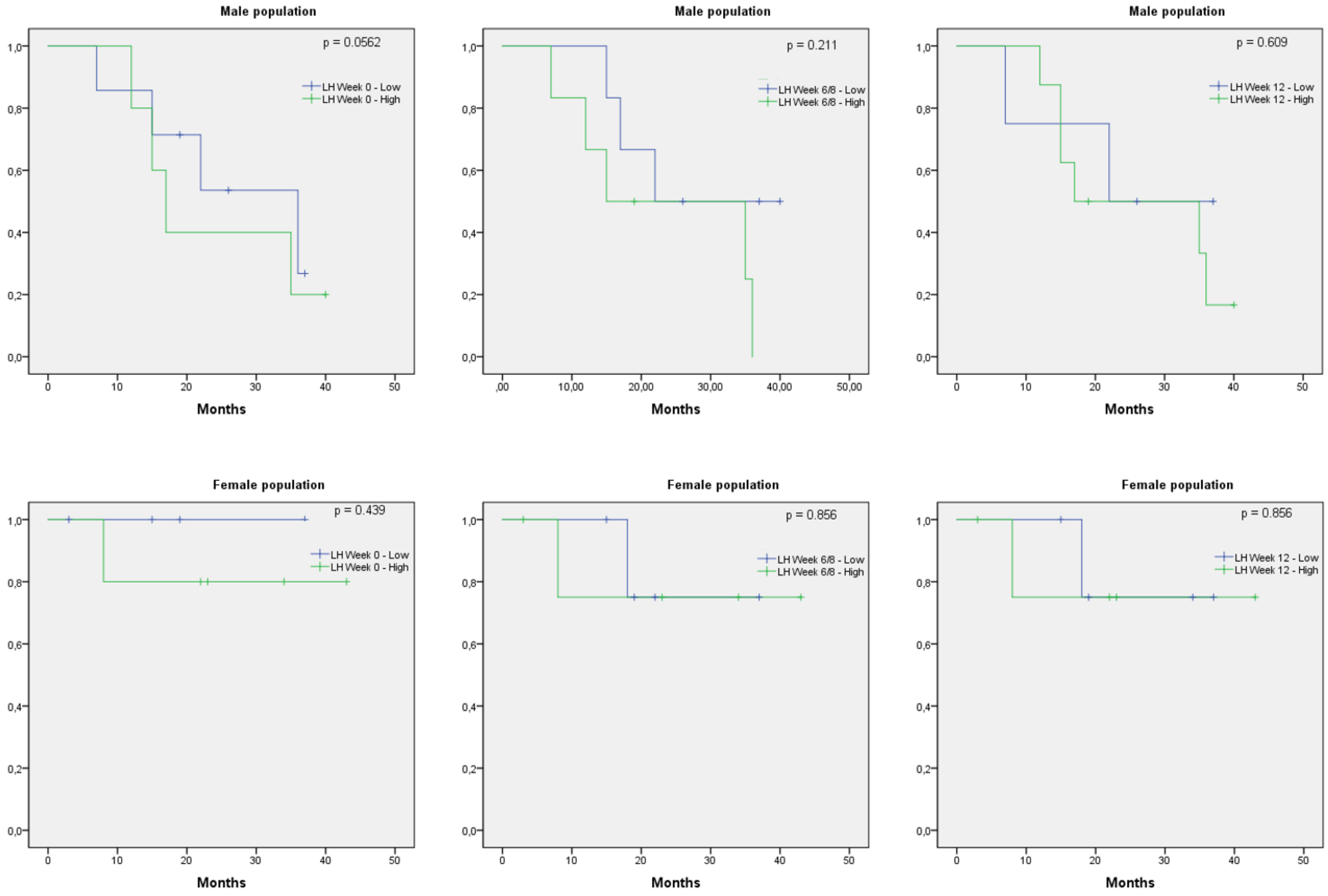

Supplementary Figure 8:
OS according to LH, dichotomized in low vs. high at the median of 6.15, 5.75 and 5.2 U/L for male population as well as at the median of 23.0, 21.4 and 24.4 U/L for female population at the three landmark evaluations performed at week 0 (baseline evaluation) 6/8 week (interim evaluation) and week 12 (final evaluation). ~~*$p < 0.05$; **$p < 0.01$; ***$p < 0.001$~~

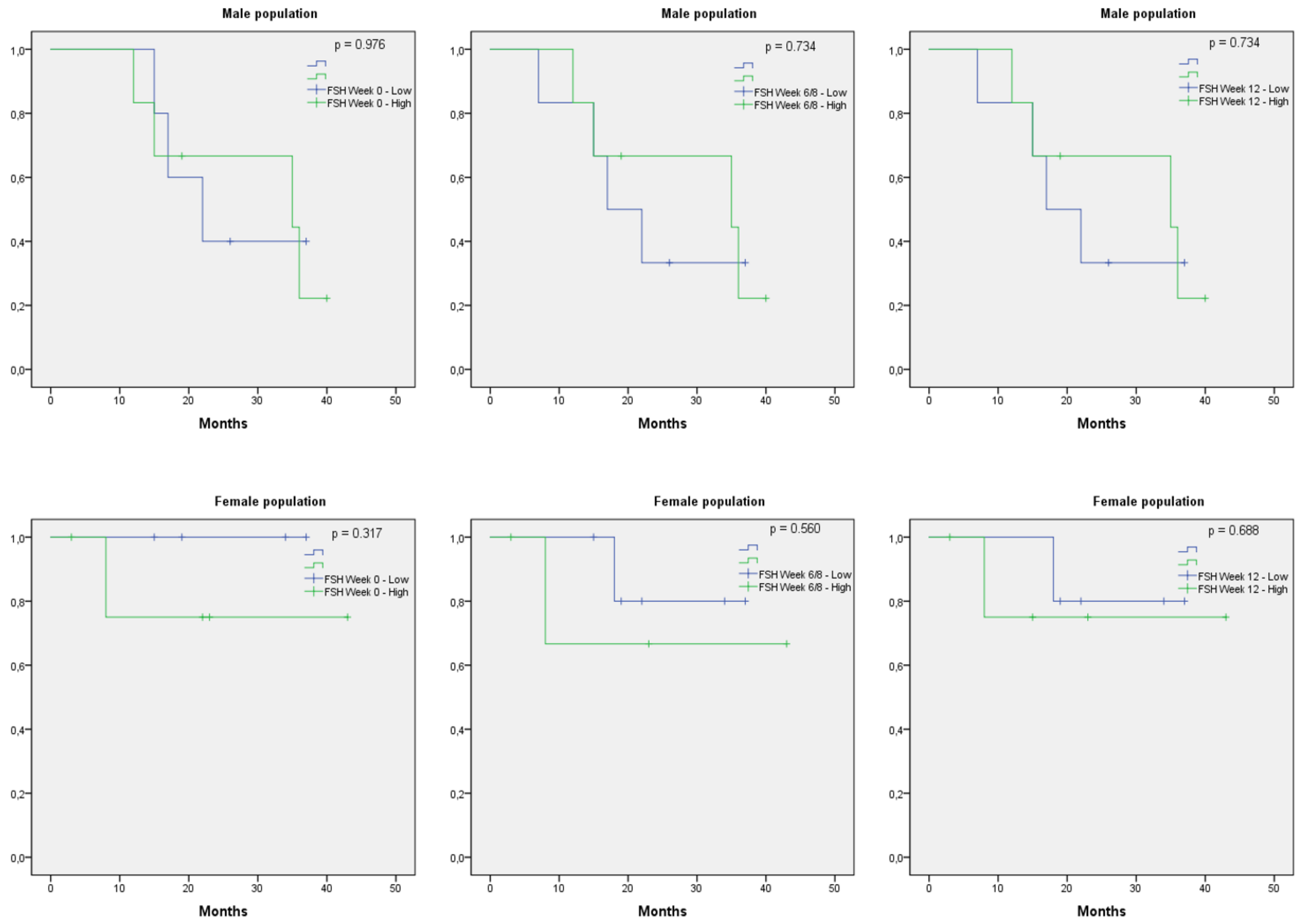

Supplementary Figure 9:
OS according to FSH, dichotomized in low vs. high at the median of 10.9, 9.3 and 8.7 U/L for male population as well as at the median of 69.4, 56.6 and 67.95 U/L for female population at the three landmark evaluations performed at week 0 (baseline evaluation) 6/8 week (interim evaluation) and week 12 (final evaluation). ~~*$p < 0.05$; **$p < 0.01$; ***$p < 0.001$~~

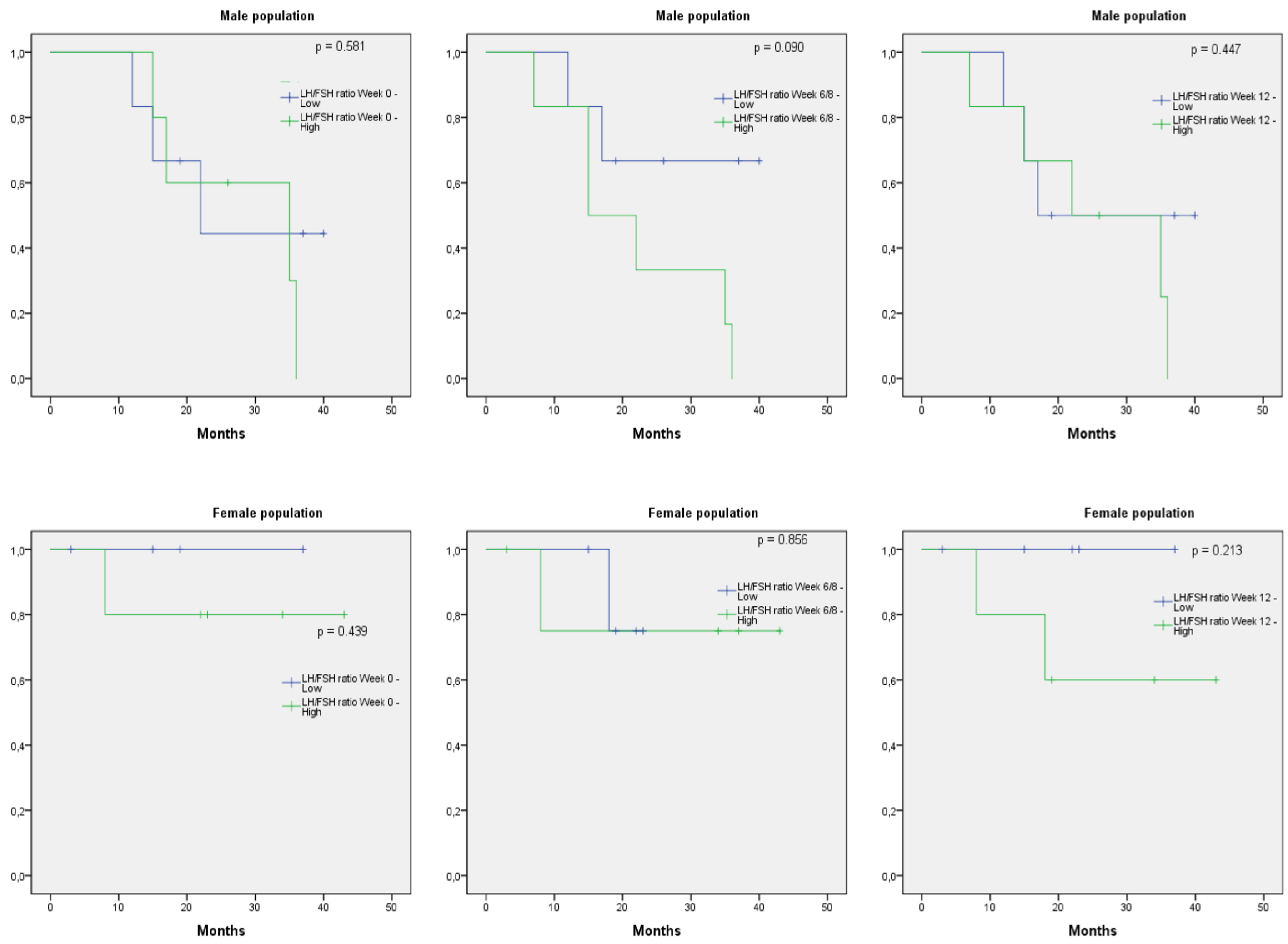

Supplementary Figure 10:
OS according to LH/FSH ratio, dichotomized in low vs. high at the median of 0.58, 0.69 and 0.72 for male population as well as at the median of 0.33, 0.36 and 0.4 for female population at the landmark evaluations performed at week 0 (baseline evaluation), 6/8 week (interim evaluation) and week 12 (final evaluation). ~~*$p < 0.05$; **$p < 0.01$; ***$p < 0.001$~~

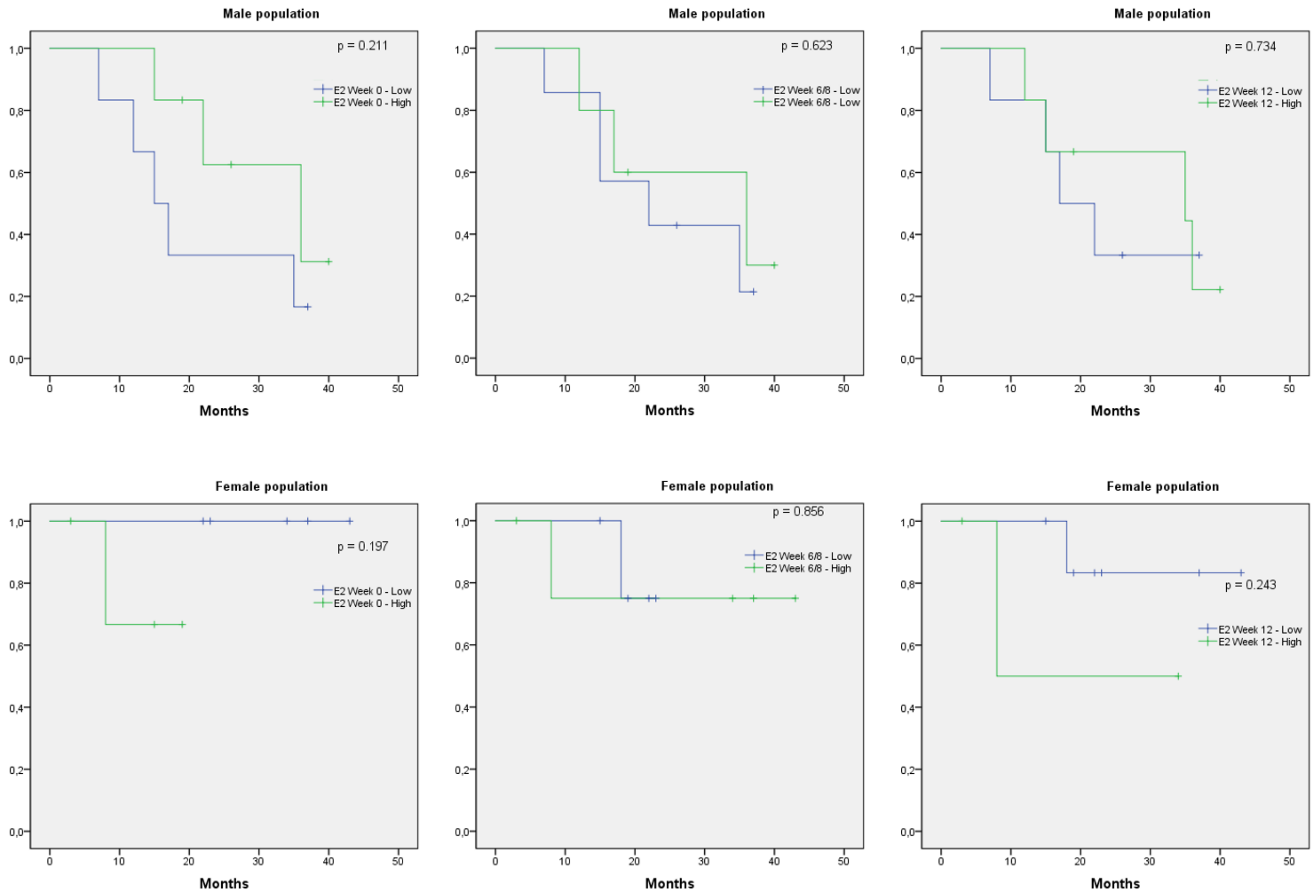

Supplementary Figure 11:
OS according to E2, dichotomized in low vs. high at the median of 21.0, 30.5 and 29 ng/ml for male population as well as at the median of 13, 13, and 13 ng/ml for female population at the three landmark evaluations performed at week 0 (baseline evaluation) 6/8 week (interim evaluation) and week 12 (final evaluation). ~~*p < 0.05; **p < 0.01; ***p < 0.001~~

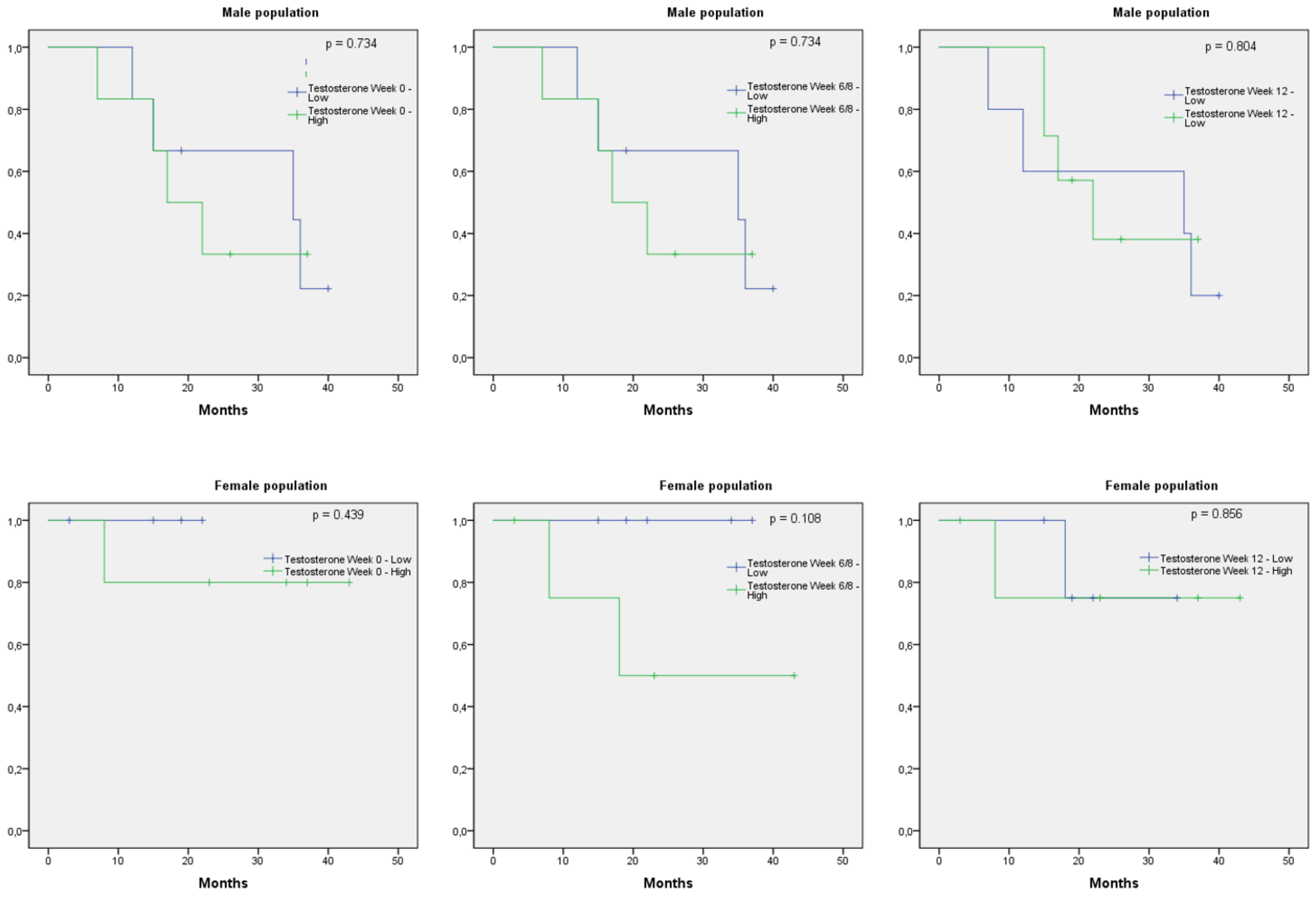

Supplementary Figure 12:
OS according to Testosterone, dichotomized in low vs. high at the median of 3.56, 4.08 and 3.58 µg/ml for male population as well as at the median of 0.14, 0.14 and 0.13 µg/ml for female population at the three landmark evaluations performed at week 0 (baseline evaluation) 6/8 week (interim evaluation) and week 12 (final evaluation). ~~*$p < 0.05$; **$p < 0.01$; ***$p < 0.001$~~

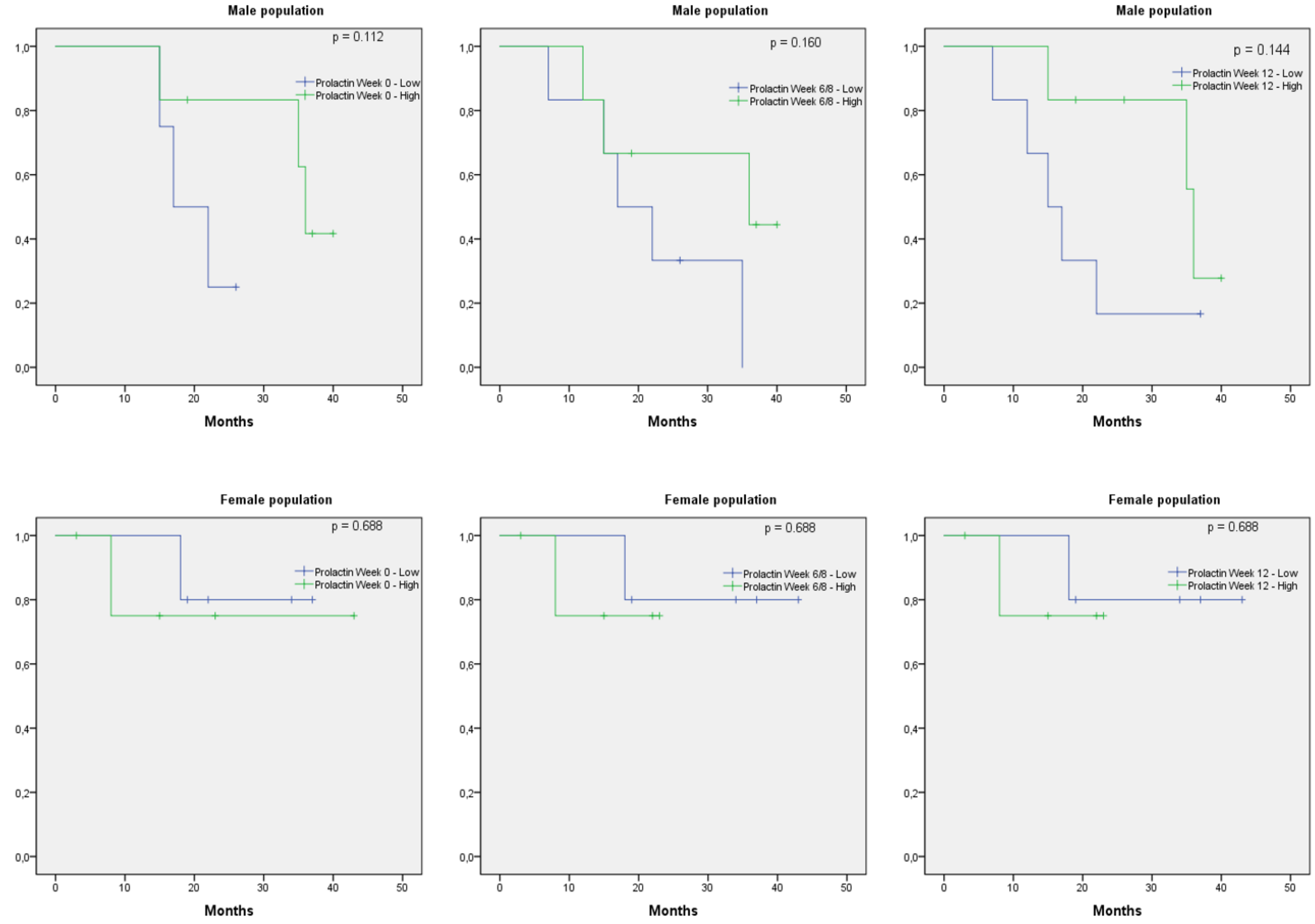

Supplementary Figure 13:
OS according to Prolactin, dichotomized in low vs. high at the median of 8.4, 10.05 and 8.7 µg/ml for male population as well as at the median of 9.5, 9.95 and 9.7 µg/ml for female population at the three landmark evaluations performed at week 0 (baseline evaluation) 6/8 week (interim evaluation) and week 12 (final evaluation). ~~*$p < 0.05$; **$p < 0.01$; ***$p < 0.001$~~

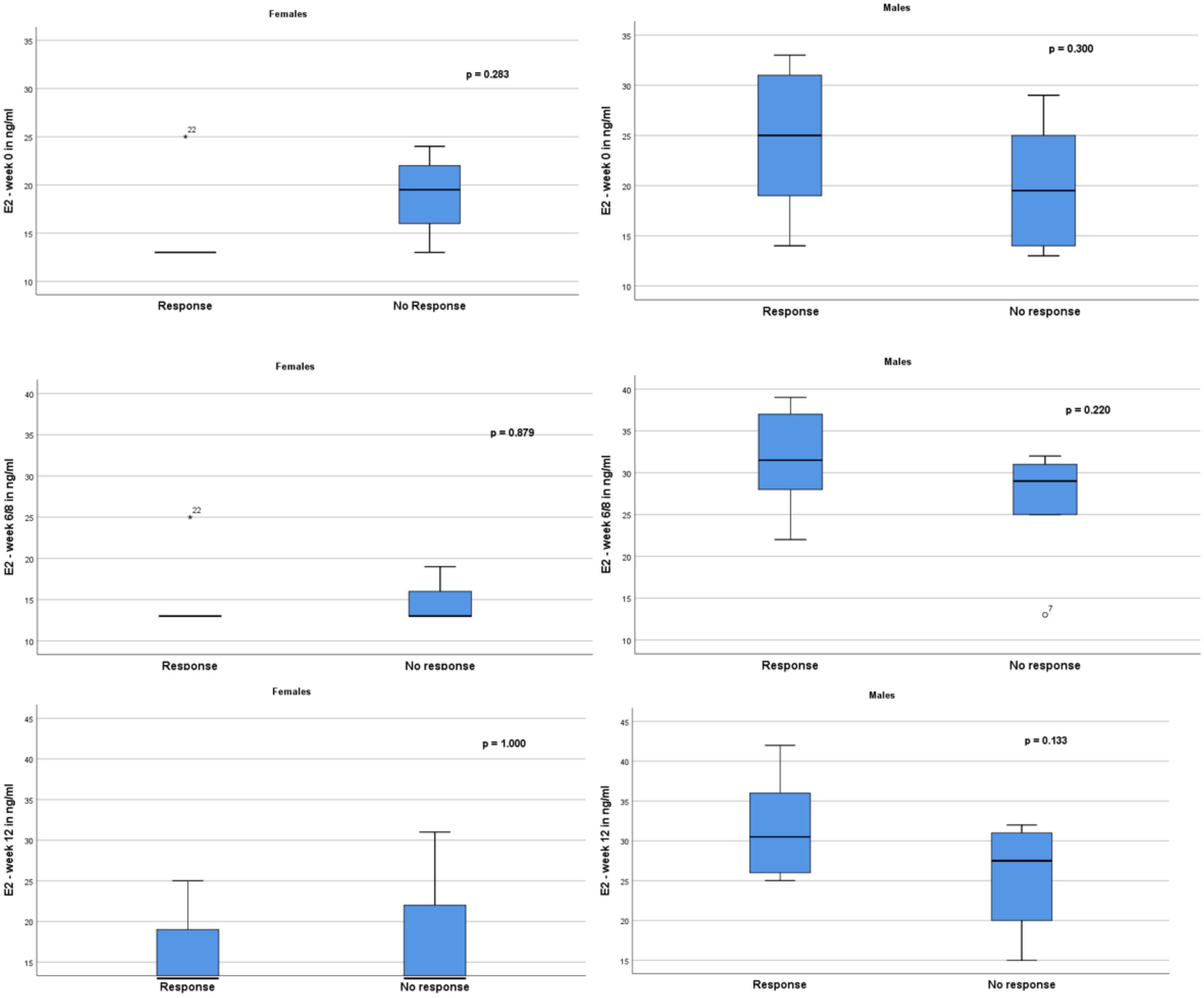

Supplementary Figure 14:
No association between E2 levels and therapy response for the male- and the female population could be shown. Therapy response was defined as SD, PR, CR by RECIST in monitoring CT after 12 weeks of treatment with nivolumab
